# Driving Precision Policy Responses to Child Health and Developmental Inequities

**DOI:** 10.1089/heq.2019.0045

**Published:** 2019-09-26

**Authors:** Sharon Goldfeld, Sarah Gray, Francisco Azpitarte, Dan Cloney, Fiona Mensah, Gerry Redmond, Katrina Williams, Sue Woolfenden, Meredith O'Connor

**Affiliations:** ^1^ Centre for Community Child Health, Murdoch Children's Research Institute, Royal Children's Hospital, Parkville, Australia.; ^2^ Department of Paediatrics, University of Melbourne, Melbourne, Australia.; ^3^ School of Social Sciences, Loughborough University, Leicestershire, United Kingdom.; ^4^ Australian Council for Educational Research, Melbourne, Australia.; ^5^ Clinical Epidemiology and Biostatistics Unit, Murdoch Children's Research Institute, Royal Children's Hospital, Melbourne, Australia.; ^6^ College of Business, Government and Law, Flinders University, Adelaide, Australia.; ^7^ Department of Neurodevelopment and Disability, Royal Children's Hospital, Melbourne, Australia.; ^8^ Clinical Sciences, Murdoch Children's Research Institute, Melbourne, Australia.; ^9^ Department of Community Child Health, Sydney Children's Hospital Network, Sydney, Australia.; ^10^ Discipline of Paediatrics, University of New South Wales, Sydney, Australia.; ^11^ ANU College of Arts & Social Sciences, The Australian National University, Canberra, Australia.

**Keywords:** inequities, child disadvantage, precision policy, evidence base

## Abstract

The growing evidence base on the extent of and opportunities to reduce inequities in children's health and development still lacks the specificity to inform clear policy decisions. A new phase of research is needed that builds on contemporary directions in precision medicine to develop precision policy making; with the aim to redress child inequities. This would include identifying effective interventions and their ideal time point(s), duration, and intensity to maximize impact. Drawing on existing data sources and innovations in epidemiology and biostatistics would be key. The economic and social gains that could be achieved from reducing child inequities are immense.

## Introduction

Inequities in children's health and development are a significant public health issue globally.^1^ These differential outcomes are unjust and preventable, and may be distinguished from inequalities (differences in health and development between population groups) by an ethical imperative.^1,2^ Health inequities arising in childhood track forward into adulthood, where they carry high costs for individuals and society, including lost human productivity and increased crime.^3^ Addressing these inequities can therefore generate substantial savings in health, education, and welfare budgets, as well as raise the productivity of society at large.^3^ Estimates from Australia suggest that if all children had the same risks of adverse outcomes as their most advantaged peers, there would be up to a 70% reduction in the incidence of poor cognitive, physical, and social–emotional outcomes.^4^

Governments (national, state, and local) have a critical role to play in reducing child inequities. This includes designing and implementing policies across multiple portfolio areas that can change the social conditions that create inequities (i.e., addressing social determinants), as well as better utilizing existing social, health, and education infrastructure to redress them.^1^ While governments in countries such as Australia include a commitment to reduce inequity in child health policy,^5^ the translation of currently available evidence into effective action continues to be a challenge.^5,6^

At the same time, we are entering the age of precision and personalization. Precision medicine is based on specific evidence about how biological factors and their interactions with each other and treatment drive patient outcomes, so that treatment (type, intensity, duration, and timing) can be adapted based on the unique profile of each patient. Precision public health approaches have focused on harnessing the power of Big Data, particularly genomic data, to inform targeted prevention approaches, including the prediction of individual risk.^7^ To make inroads on health inequities, however, as well as considering programs and services aimed at the individual or micro level, there is a need to remain steadfastly focused on interventions and reforms that impact the institutions, social systems, and public policies that fundamentally drive health inequities.^7,8^

To this end, we argue for the need to apply precision concepts to developing evidence that can enable *precision policy making*. This would require specificity in the evidence about modifiable factors that redress child inequities; including evidence about their interactions with each other and existing interventions to create real world synergistic solutions. Equipped with this evidence, policy makers can choose the maximally effective public health, social, education, or health services strategy (or more likely series of strategies), target populations of children, developmental timing, and intensity and duration to reduce child inequities.^4^ Accelerating our research in this new direction will require wide ranging and trans-disciplinary collaborations, while maximizing the potential of existing evidence and data sources.

There are successful examples internationally where researchers have helped to inform precise policy decisions and action through evidence generation. For example, research from the United States found that children whose parents work unpredictable schedules or nonstandard hours exhibit poorer cognitive and behavioral outcomes, with disadvantaged and minority children being disproportionately impacted.^9^ A clear implication of these findings was the need to create disincentives for employers to schedule work hours in a way that impacts on parenting capacities. This has since been actioned through legislation to address unpredictable work scheduling practices.^9^

## What Is Known Already?

**FIG. 1.**
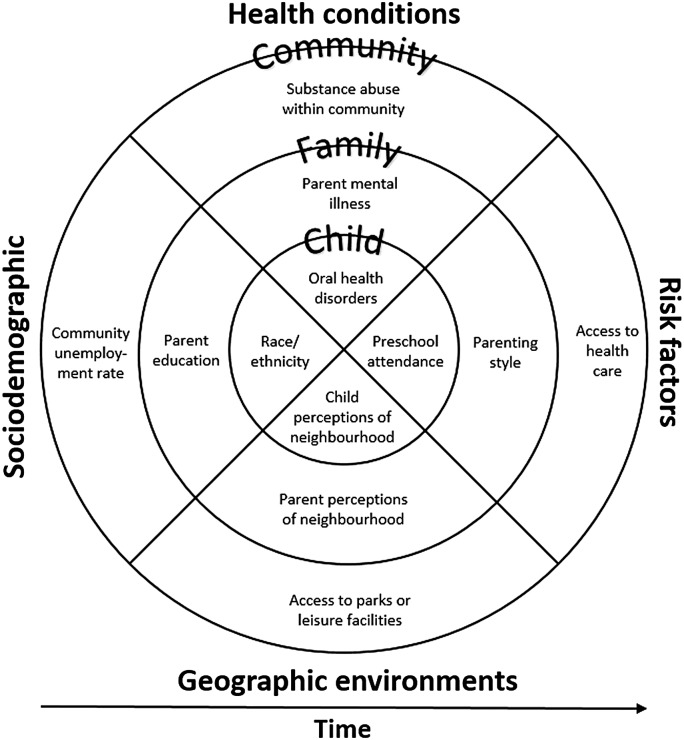
Framework of child disadvantage reproduced from Goldfeld et al. aligning a social determinants and bio-ecological perspective. Examples of relevant indicators within each lens (sociodemographic, geographic environments, health conditions, and risk factors) and level (child, family, and community) are shown. It is expected that disadvantage experienced through each of these lenses will overlap and interact to influence inequities in complex ways.

Existing research has laid a strong foundation for building the evidence base to inform precise action to reduce child inequities. Disadvantage refers to relative position in a social hierarchy determined by wealth, power, and prestige.^10^ Relative disadvantage is multidimensional, and manifests across the circumstances in which children, live, learn, and develop (i.e., social determinants).^11^ Disadvantage can be experienced through four social determinants lenses (Fig. 1), which can overlap and change over time.^11^

**FIG. 2.**
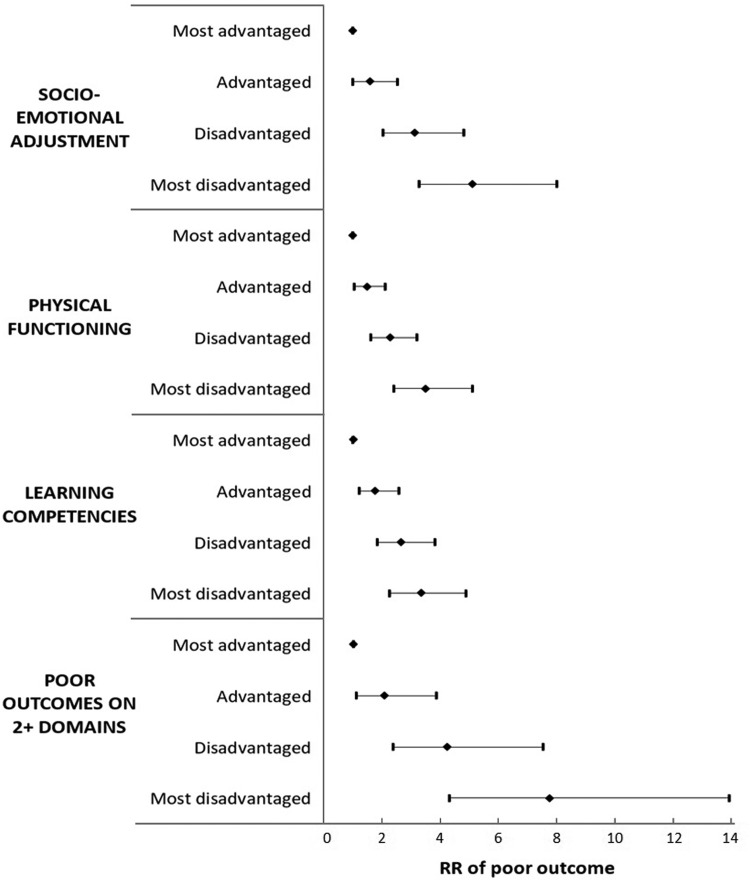
Relative risk of having poor developmental outcomes (bottom 15%) at age 10–11 years associated with disadvantage trajectories over childhood. Data originally presented in Goldfeld et al.^4^

Evidence to date shows starkly that inequities exist in all countries across children's physical health, social–emotional, and cognitive outcomes.^1^ In Australia, for example, children on a persistently disadvantaged pathway over early childhood have a sevenfold increased risk of having poor outcomes in multiple developmental domains by late childhood, compared to the most advantaged children (Fig. 2).^4^ Inequities affect a much larger proportion of the population than just those who are most severely disadvantaged (i.e., experiencing poverty); children experience poorer outcomes for each increment of increasing disadvantage.

There is strong evidence to suggest that intervening in early childhood—such as through good quality preschool,^12^ optimizing the quality of parenting and the home learning environment,^13^ cash transfers,^14^ and mutually reinforcing combinations of strategies^15^—is the most effective and cost effective means of reducing the societal burden of health inequities.^3^ However, our current policy efforts in these areas are not closing these gaps.^13^ Governments continue to make large social service investments to remedy inequities later in life (e.g., job training, crime rehabilitation), rather than having a comprehensive policy and prevention funding response in the early years.^3^

## What Evidence Is Needed?

Evidence needed to inform the development of precision policy to redress child inequities includes identifying effective interventions to target specific populations of children, and the time point(s), duration and intensity, and intervention combinations that can maximize impact (Table 1). The continued development of ethical frameworks for defining and measuring inequities, as distinct from inequalities, is also an important research goal.^2^

**Table 1.** Examples of Research Questions that Need Addressing in the Next Phase of Child Inequity Research

Research area	Specific topics/questions
Defining priorities	How should inequities be defined? Which inequalities are just and acceptable and which are not; and which inequalities are modifiable?
	How should we prioritize addressing inequities across different child outcomes and contexts?
Intervention targets	Of the multiple potential or known modifiable leverage points for intervention, which have the greatest potential to reduce inequities in children's outcomes?
Combinations of strategies	Given that interventions may be most effective when they are multi-pronged and reinforced over time, which intervention combinations will have the greatest impact?
	What combinations of supports across settings (e.g., school, home, and built environment) are most effective?
Timing and dosage of strategies	At which point in development, and at what dosage, would identified combinations of interventions achieve the greatest gain in child outcomes?
Populations of children to target	Which delivery approach/s are likely to have the greatest impact on reducing inequities in children's outcomes (e.g., universal, targeted)?
	Which subpopulations might benefit most from targeted strategies?
	How do the effects of interventions vary for children from different population groups, or for children who differ on a relevant determinant (e.g., different levels of parent education)?
Outcomes impacted	For the above, in what specific domain/s of child development is there an effect?

Preschool is one example of where greater precision in the evidence is needed. Many governments see preschool as a major opportunity to reduce inequities. While current evidence suggests that preschool may be effective for certain populations,^12^ in the Australian context there is a lack of evidence to inform with any level of specificity what approach is most effective.^16^ In the absence of this evidence, policy is being enacted regardless, such as decisions on the number of hours of preschool attendance to subsidize.

In contrast, precision policy responses could draw on specific evidence about what is most effective in terms of number of hours; curriculum/program elements most predictive of outcomes; teacher qualification; duration (i.e., 1 year vs. 2 years); complementarity with other programs and supports; and approaches to engaging the most vulnerable families.^16^ The development of such evidence would be informed by input from policy agencies and practitioners to ensure that the design reflects the current real-world considerations. Once findings were available, policy makers would inform interpretation and jointly consider where effort might be strategically and practically placed to effect recommendations for action.

## Next Steps in Developing the Evidence Base for Precision Policy

Addressing the questions highlighted in Table 1 will require drawing together different strands of relevant evidence across multiple disciplines (e.g., from genomics to social determinants), and synthesizing findings across study designs, including observational studies, systematic reviews, and randomized controlled trials (RCTs). While recognizing the important role these designs each play, we emphasize here the opportunities afforded by population cohort studies.^17,18^ Increasing data volume and variety in epidemiology and the expansion of “big data” have made possible the precision medicine and precision public health movements.^18^ The breadth of longitudinal and multilevel data available in existing cohorts, maximized through linkage with administrative data, can similarly enable researchers to generate policy relevant findings quickly and cost effectively. Well considered and comprehensive analyses of cohort data can provide robust evidence on opportunities and potential to reduce child inequities that can guide decision making, including where to target further evaluative intervention studies.^19^ Future cohorts may well provide the platform for within cohort trials in a more integrated approach to develop the evidence for precision thinking.

Utilizing secondary data in innovative ways requires careful thought and planning to address analytic challenges like attrition of those most disadvantaged from the cohorts over time.^17^ Notwithstanding these challenges, through choice of analytic methods it is possible to use cohort data to go beyond examining associations between exposures and outcomes to make the stronger inferences about causal relationships that are required to meaningfully inform policy. For instance, techniques such as causal mediation continue to be advanced^20^; while techniques such as marginal structural models make it feasible to simulate what intervention can realistically achieve.^21^ Techniques such as effect-measure modification can help to assess whether an intervention may have varying effects for different subgroups of children (e.g., ethnic minority groups).^22^

When designing analyses, researchers require a nuanced complex systems perspective.^7^ This includes accounting for the many interrelated factors influencing child health and development and their temporal ordering; considering the importance of timing of different exposures in determining the significance of their impact.^23^ Failure to do so presents a risk of treating single risk factors as if they occur in a vacuum, with the implication being narrow or isolated “magic bullet” intervention strategies with limited long-term impact.^24^

In addition to such investigations in cohort data, larger data sources enable precision for subpopulations. For example, routine antenatal care data have informed how the Australian health care system can be improved for women of refugee backgrounds, which would be challenging to capture within mainstream cohorts.^25^ RCTs have the further potential for innovative policy relevant designs, such as stepped wedge.^26^ In addition to making best use of existing data, we should also make best use of existing evidence, utilizing systematic reviews addressing social determinant risk factors from longitudinal population studies and policy relevant interventions.^27,28^

Ethical concerns have been raised regarding precision medicine that aim to identify individuals or populations “at risk”; such as privacy, and discrimination and stigmatization.^18^ How to weigh up the goal of addressing inequities with the need to maximize overall public benefit is also complex; for example, when it is less cost effective to improve the outcomes of those who are the very worst off, than to improve outcomes of those slightly better off.^29^ Managing these concerns will be vital for researchers and policymakers seeking to build the evidence base.

The ultimate translation of this work into precision policy is not without its complexities. Even when evidence carries clear and precise policy implications, hurdles remain in the way of policy action, including competing political interests, resource and time constraints, and siloing of government portfolios.^6^ Even the best evidence guided interventions may not work as intended,^30^ highlighting the need for continued evaluation and monitoring of policy efforts. As this new thinking enabled by recent methodological advances is implemented from concept to policy change, it will be vital to document case studies and exemplars to learn from and show what is possible, and evaluate this process itself.

## Conclusion

Research designed to address or prevent child health inequity needs to enter a new phase of precision. The challenge is to develop ethical frameworks to define and prioritize inequities, and to build an evidence base with sufficient specificity to generate actionable policy implications capable of leading to measurable change. Greater precision in policy will help to direct limited public funds toward intervention opportunities that will have the greatest impact. This ensures that resources are put to the best possible use and maximizes return on investment. Redressing child inequities is both a social justice and economic imperative.

## Abbreviations Used

NHMRC: National Health and Medical Research Council
RCT: randomized controlled trial
